# Subclinical joint inflammation in rheumatoid arthritis: comparing thermal and ultrasound imaging at the metacarpophalangeal joint

**DOI:** 10.1186/s42358-024-00377-9

**Published:** 2024-05-03

**Authors:** York Kiat Tan, Gek Hsiang Lim

**Affiliations:** 1 Department of Rheumatology and Immunology, Singapore General Hospital, Outram Road, Bukit Merah, Central Region, 169608 Singapore; 2 Duke-NUS Medical School, Singapore, Singapore; 3 Yong Loo Lin School of Medicine, National University of Singapore, Queenstown, Singapore; 4 Health Services Research Unit, Singapore General Hospital, Bukit Merah, Central Region, Singapore

**Keywords:** Thermography, Ultrasonography, Rheumatoid arthritis, Joints, Synovitis

## Abstract

**Background:**

While ultrasound and MRI are both superior to clinical examination in the detection of joint inflammation, there is presently a lack of data whether thermography may be similarly useful in the assessment of joint inflammation in patients with RA. Our study aims to evaluate the use of thermography in detecting subclinical joint inflammation at clinically quiescent (non-tender and non-swollen) metacarpophalangeal joints (MCPJs) in patients with rheumatoid arthritis (RA). The outcomes from thermography in our study will be compared with ultrasonography (which is a more established imaging tool used for joint inflammation assessment in RA).

**Methods:**

The minimum (Tmin), average (Tavg) and maximum (Tmax) temperatures at the 10 MCPJs of each patient were summed to obtain the Total Tmin, Total Tavg and Total Tmax, respectively. Ultrasound grey-scale (GS) and power Doppler (PD) joint inflammation (scored semi-quantitatively, 0–3) at the 10 MCPJs were summed up to derive the respective TGS and TPD scores per patient. Pearson’s correlation and simple linear regression were respectively used to assess correlation and characterize relationships between thermographic parameters (Total Tmin, Total Tavg and Total Tmax) and ultrasound imaging parameters (TGS, TPD and the number of joint(s) with PD ≥ 1 or GS ≥ 2).

**Results:**

In this cross-sectional study, 420 clinically non-swollen and non-tender MCPJs from 42 RA patients were examined. All thermographic parameters (Total Tmin, Total Tavg and Total Tmax) correlated significantly (*P*-values ranging from 0.001 to 0.0012) with TGS score (correlation coefficient ranging from 0.421 to 0.430), TPD score (correlation coefficient ranging from 0.383 to 0.424), and the number of joint(s) with PD ≥ 1 or GS ≥ 2 (correlation coefficient ranging from 0.447 to 0.465). Similarly, simple linear regression demonstrated a statistically significant relationship (*P*-values ranging from 0.001 to 0.005) between all thermographic parameters (Total Tmin, Total Tavg and Total Tmax) and ultrasound imaging parameters (TPD and TGS).

**Conclusion:**

For the first time, thermographic temperatures were shown to correlate with ultrasound-detected joint inflammation at clinically quiescent MCPJs. The use of thermography in the detection of subclinical joint inflammation in RA appears promising and warrants further investigation.

## Introduction

Thermography, an emerging imaging technique, can offer a quick and objective measurement of joint surface temperature in patients with rheumatoid arthritis (RA). Thermal imaging has high feasibility for use, as it is a safe and non-invasive imaging modality, with modern thermal cameras being compact, highly portable and easy to use [1]. Therefore, thermography is well-suited for use in the Rheumatologist’s office as an adjunctive tool for joint inflammation assessment in patients with RA [2]. Apart from being convenient for use, thermography is also a contactless imaging modality. Hence, it can potentially be developed as a tool to aid joint inflammation assessment for remote telemedicine consultations whereby direct physical examination of the joint sites cannot be carried out [3]. Conventionally, Rheumatologists assess joint inflammation by eliciting the absence or presence of joint swelling and tenderness [4, 5] through clinical joint examination. The tender joint count (TJC) and swollen joint count (SJC) are physician-elicited clinical assessment outcomes which are core components of the 28-joint disease activity score (DAS28) and the American College of Rheumatology (ACR) response criteria, both of which are commonly used as RA disease activity measures [4–6]. In the past two decades, modern imaging tools such as ultrasound and magnetic resonance imaging (MRI) have taught us valuable lessons in the assessment of joint inflammation in patients with RA/inflammatory arthritis. Specifically, both ultrasound and MRI are known to be more sensitive than clinical examination in detecting joint inflammation [7–10]. For example, in a study involving 80 untreated oligoarthritis patients, ultrasound detected synovitis in 150 out of 459 (33%) joints that were deemed clinically not to have synovitis [9]. In another small scale study comparing MRI with clinical examination at the hands and wrists of active RA patients, it was found that MRI detected synovitis significantly more frequently than clinical examination (162 versus 59 joints; *p* = 0.00002) [10], particularly for the MCPJ and proximal interphalangeal joint (PIPJ). Given the superiority of ultrasound and MRI over clinical examination, the European Alliance of Associations for Rheumatology (EULAR) has included in its recommendations that ultrasound (and MRI) should be considered for more accurate assessment of joint inflammation and these imaging modalities may be useful in monitoring disease activity in patients with RA [11]. However, the use of ultrasound and MRI are not without limitations [12, 13]. For example, the former may be time-consuming especially when scanning multiple joint sites and a considerable period of training will be required for sonographers to attain proficiency, while the latter is generally costly which can be prohibitive for widespread use and there are specific magnet-related contraindications (e.g. pacemakers, etc.). Given these limitations, it is therefore necessary to explore the use of other imaging modalities such as thermography which has high feasibility for use (e.g. low cost, safe, quick and convenient to use). While ultrasound and MRI are both superior to clinical examination in the detection of joint inflammation, there is presently a dearth of knowledge on whether thermography may be similarly useful in the assessment of joint inflammation in patients with RA. Therefore, to address this knowledge gap, our study aims to evaluate the use of thermography in detecting subclinical joint inflammation at clinically quiescent (non-tender and non-swollen) metacarpophalangeal joints (MCPJs) in patients with rheumatoid arthritis (RA). The outcomes from thermography in our study will be compared with ultrasonography (which is a more established imaging tool used for joint inflammation assessment in RA).

## Methods

This is a single site cross-sectional study, whereby patients with RA (fulfilling the 2010 EULAR/ACR) [14] with clinically quiescent (non-tender and non-swollen) MCPJs were consecutively recruited between December 2020 and June 2023 from the outpatient clinic of the Rheumatology unit at a tertiary care local hospital. Our study was approved by our local institutional review board and conforms to the relevant research ethical guidelines. All patients recruited into our study provided their informed consent prior to enrolment.

### Baseline characteristics of the patients

The following baseline characteristics of the patients were obtained from the hospital medical records: age; ethnicity; gender; duration of disease; underlying disease-modifying anti-rheumatic drugs (DMARDs) and corticosteroid use. Baseline DAS28 was performed by trained nurses from the Rheumatology unit (blinded to the findings from thermal and ultrasound imaging) on the same day as the thermography and ultrasonography.

### Imaging assessments

Ultrasonography was performed by a single rheumatologist experienced in musculoskeletal ultrasound imaging, while a separate study personnel carried out the thermography while being blinded to the outcomes from the ultrasound imaging. Following published EULAR guidelines [15], standardized ultrasound imaging was carried out using the Mindray M9 ultrasound machine (and L14-6Ns linear probe) with machine settings as follows: pulse repetition frequency (PRF) at 700 Hz and Doppler frequency at 5.7 MHz. Ultrasound grey-scale (GS) synovial hypertrophy and power Doppler (PD) were graded semi-quantitatively (using a 0–3 severity scale, i.e. 0 = none, 1 = mild, 2 = moderate and 3 = severe) based on previous established methods with acceptable inter/intra-observer reliability [16, 17].

Standardized thermal imaging was conducted following previously established methods described in the literature [1, 2, 18–20]. As per standard practice, the enrolled patients were rested 15 min (to facilitate acclimatization) before beginning thermal imaging [2, 19]. Thermography was carried out in a draft-free room (with no windows) at an ambient temperature of about 23 °C [18] using the FLIR T640 high performance portable thermal camera. The settings of the thermal camera were as follows: predefined emissivity value of 0.98 for skin [1, 2 and 20]. pixel resolution of 640 × 480 and thermal sensitivity of < 30 milli-Kelvin at 30 °C. The MCPJs of each hand were imaged in a standardized manner with the thermal camera held 50 cm above the dorsum of the hand placed on a flat surface.

Using the commonly utilized region of interest (ROI) manual segmentation approach [1, 2, 19 and 20], the target ROIs on the grey-scale images was selected by placing a rectangular box over the anatomical sites (i.e. the MCPJs). Finally, the minimum (Tmin), average (Tavg) and maximum (Tmax) temperatures (in °C) were recorded from each MCPJ ROI.

### Statistical analysis

The Tmin, Tavg and Tmax temperatures at the 10 MCPJs of each patient were summed up to obtain the Total Tmin, Total Tavg and Total Tmax, respectively. Similarly, the ultrasound GS and PD sub-scores at the 10 MCPJs were summed up to obtain the Total GS (TGS) and Total PD (TPD) scores respectively. Ultrasound synovitis at each MCPJ is defined as PD ≥ 1 or GS ≥ 2 [21–24]. Pearson’s correlation was used for correlation analysis between thermographic parameters (Total Tmin, Total Tavg and Total Tmax) and ultrasound imaging parameters (TGS, TPD and the number of joint(s) with PD ≥ 1 or GS ≥ 2). Simple linear regression was used to assess correlation and characterize relationships of thermographic parameters (Total Tmin, Total Tavg and Total Tmax) and ultrasound imaging parameters (TGS, TPD and the number of joint(s) with PD ≥ 1 or GS ≥ 2). Statistical significance is declared if a two-sided *p*-value is less than 0.05. The statistical analyses were performed using Stata 17 (StataCorp. 2021. *Stata Statistical Software: Release 17*. College Station, TX: StataCorp LLC).

## Results

### Baseline characteristics of the patients

A total of 420 clinically non-swollen and non-tender MCPJs from 42 RA patients were assessed in this study. The mean (SD) age of the study population is 57.7 (13.2) years. Out of the 42 patients, 35 (83.3%) are female, and 31 (73.8%) are Chinese. The mean (SD) disease duration and baseline DAS28 of the patients are 38.2 (59.2) months and 3.10 (0.82) respectively. At the time of recruitment, 25/42 patients (59.5%) were on oral prednisolone, and all patients (100%) were on one or more oral conventional DMARDs (methotrexate, leflunomide, sulfasalazine and hydroxychloroquine).

### Correlation between thermal and ultrasound imaging parameters

Table 1 summarises the results from the correlation analysis between the thermal and ultrasound imaging parameters. For ultrasound GS joint inflammation, all thermographic parameters (Total Tmin, Total Tavg and Total Tmax) correlated significantly (*P*-values ranging from 0.005 to 0.006) with TGS scores (Pearson’s correlation coefficient ranging from 0.421 to 0.430). For ultrasound PD joint inflammation, all thermographic parameters (Total Tmin, Total Tavg and Total Tmax) correlated significantly (*P*-values ranging from 0.001 to 0.012) with TPD scores (Pearson’s correlation coefficient ranging from 0.383 to 0.424). For joint(s) displaying ultrasound synovitis (PD ≥ 1 or GS ≥ 2), all thermographic parameters (Total Tmin, Total Tavg and Total Tmax) correlated significantly (*P*-values ranging from 0.002 to 0.003) with the number of joint(s) with PD ≥ 1 or GS ≥ 2 (Pearson’s correlation coefficient ranging from 0.447 to 0.465).

**Table 1:** Result of correlation analysis between the thermal and ultrasound imaging parameters

Thermographic parameter	Total GS score		Total PD score		Number of joint(s) with PD ≥1 or GS ≥2	
	Pearson’s Correlation coefficient	*P*-value	Pearson’s Correlation coefficient	*P*-value	Pearson’s Correlation coefficient	*P*-value
Total Tmin	0.430	0.005**	0.383	0.012*	0.447	0.003**
Total Tavg	0.424	0.005**	0.395	0.001**	0.451	0.003**
Total Tmax	0.421	0.006**	0.424	0.005**	0.465	0.002**

Abbreviations: Tmin, minimum temperature; Tavg, average temperature; Tmax, maximum Temperature. Statistically significant: *P < 0.05, **P < 0.01.

### Linear regression analysis

**Fig. 1:**
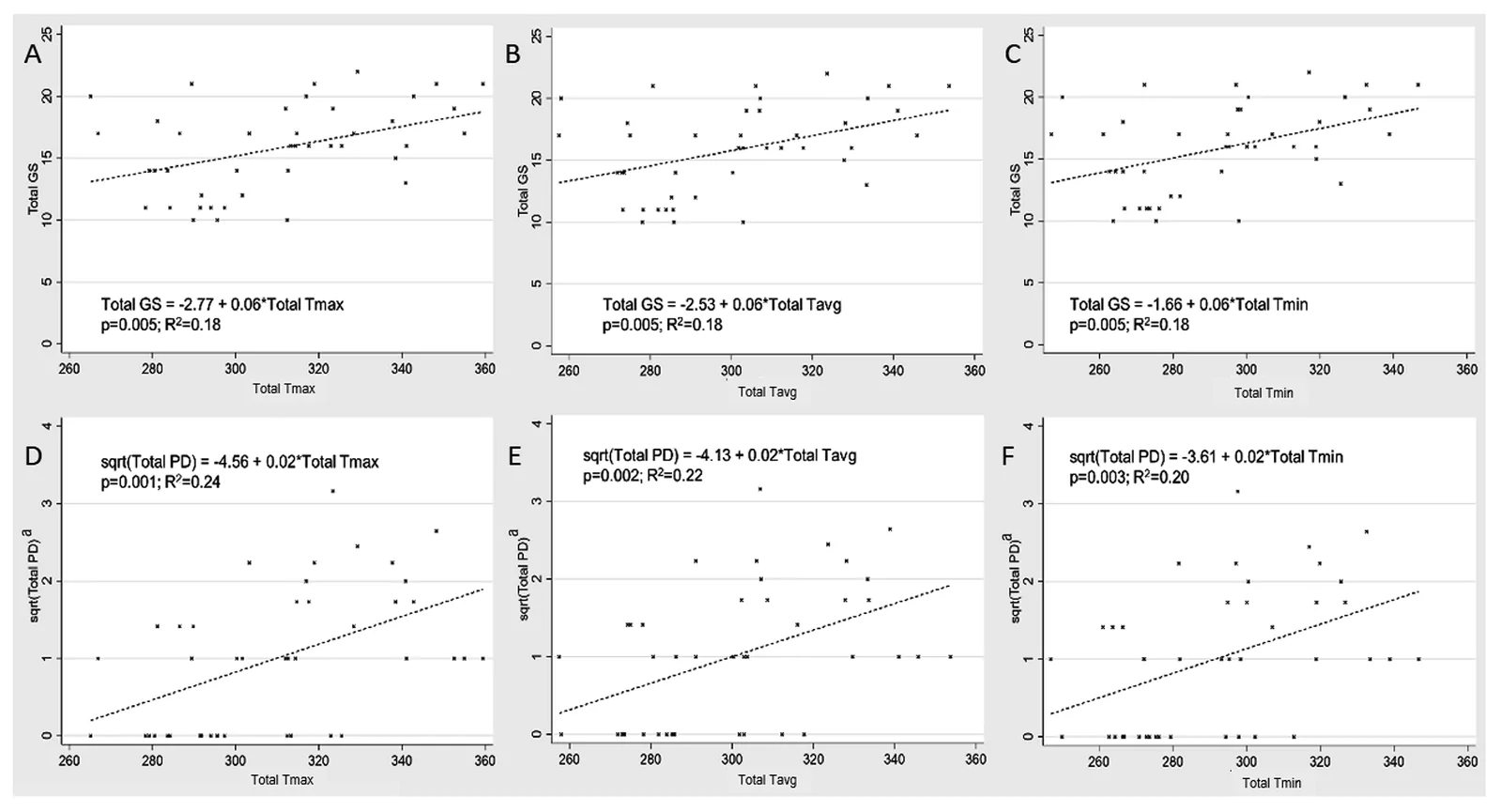
Relationship between thermographic and ultrasound imaging (Total GS and Total PD) parameters. **A**-**C** Thermographic parameters versus Total GS. **D**-**F** Thermographic parameters versus Total PD. Abbreviations: GS, grey-scale; PD, power Doppler; Tmax, maximum temperature; Tavg, average temperature; Tmin, minimum temperature. ^a^Total PD was transformed to square root (sqrt) Total PD to normalise its distribution

**Fig. 2:**
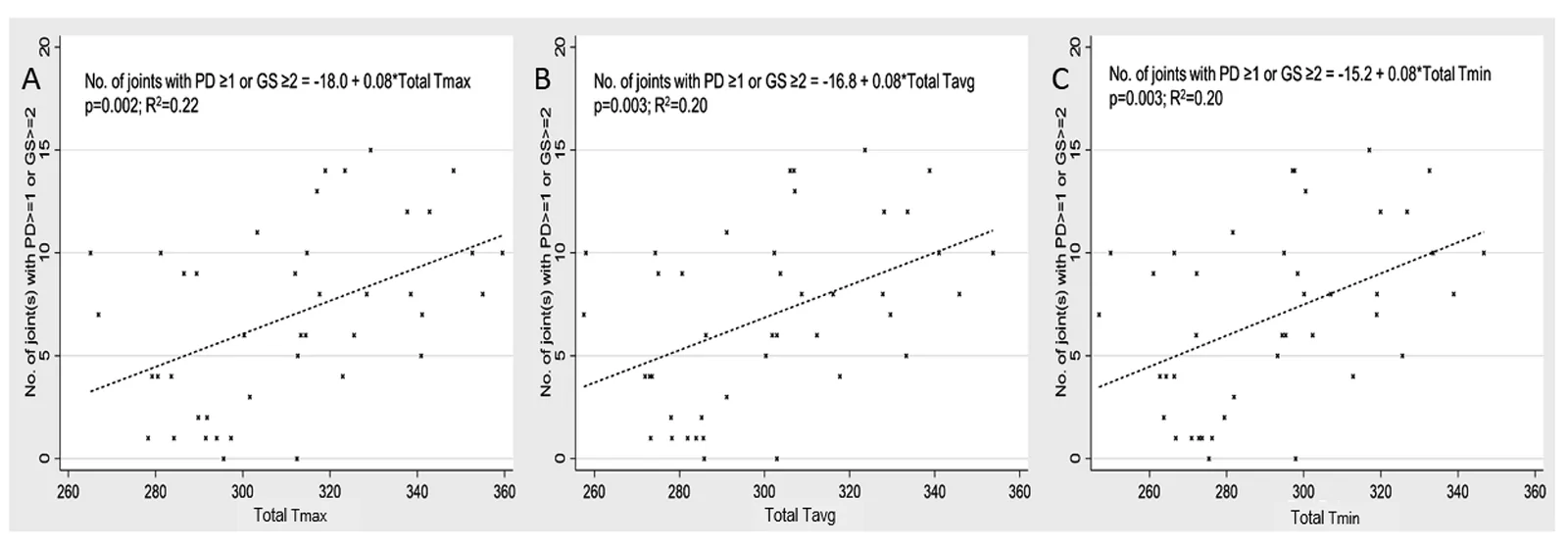
Relationship between thermographic parameters and number of joint(s) with PD ≥ 1 or GS ≥ 2. A-C Thermographic parameters versus number of joint(s) with ultrasound synovitis (PD ≥ 1 or GS ≥ 2). Abbreviations: PD, power Doppler; GS, grey-scale; Tmax, maximum temperature; Tavg, average temperature; Tmin, minimum temperature

For ultrasound GS joint inflammation, linear regression analysis demonstrated a statistically significant relationship (Fig. 1) between all thermographic parameters (Total Tmin, Total Tavg and Total Tmax) and TGS score as follows: TGS versus Total Tmin (R^2^ = 0.18, *P* = 0.005); TGS versus Total Tavg (R^2^ = 0.18, *P* = 0.005); TGS versus Total Tmax (R^2^ = 0.18, *P* = 0.005). For ultrasound PD joint inflammation, linear regression analysis demonstrated a statistically significant relationship (Fig. 1) between all thermographic parameters (Total Tmin, Total Tavg and Total Tmax) and TPD score (note: TPD was transformed to square root (sqrt) TPD to normalise its distribution) as follows: sqrtTPD versus Total Tmin (R^2^ = 0.20, *P* = 0.003); sqrtTPD versus Total Tavg (R^2^ = 0.22, *P* = 0.002); sqrtTPD versus Total Tmax (R^2^ = 0.24, *P* = 0.001). For joint(s) displaying ultrasound synovitis (PD ≥ 1 or GS ≥ 2), linear regression analysis demonstrated a statistically significant relationship (Fig. 2) between all thermographic parameters (Total Tmin, Total Tavg and Total Tmax) and the number of joints with PD ≥ 1 or GS ≥ 2 as follows: number of joint(s) with PD ≥ 1 or GS ≥ 2 versus Total Tmin (R^2^ = 0.20, *P* = 0.003); number of joint(s) with PD ≥ 1 or GS ≥ 2 versus Total Tavg (R^2^ = 0.20, *P* = 0.003); number of joint(s) with PD ≥ 1 or GS ≥ 2 TGS versus Total Tmax (R^2^ = 0.22, *P* = 0.002).

## Discussion

While ultrasound and MRI have superiority over clinical examination and can help detect subclinical joint inflammation [7, 8], data on thermographic detection of subclinical joint disease in RA is presently lacking. For the first time, our study has demonstrated that thermographic temperatures are associated with ultrasound-detected joint inflammation at clinically quiescent (non-tender and non-swollen) MCPJs. Specifically, all our studied thermographic parameters (Total Tmin, Total Tavg and Total Tmax) showed significant correlation with ultrasound-detected joint inflammation outcomes (TGS, TPD and number of joint(s) with ultrasound synovitis defined as PD ≥ 1 or GS ≥ 2). In the past decade, there has been increased interest in the use of thermography as an assessment tool in patients with inflammatory and degenerative joint conditions based on publication trend [25]. Among the various joint sites examined by thermography, the hand is the most frequently studied anatomical site [1], and differences in thermographic findings have been observed between RA patients and healthy controls [26–29]. To the best of our knowledge, no previous RA studies have specifically looked at subclinical joint inflammation detection correlating thermographic temperatures with ultrasound-detected joint inflammation outcomes. One previous small-scale cross-sectional study by Tan et al. [30] examined thermal and ultrasound imaging at the bilateral MCPJs, PIPJs, thumb inter-phalangeal joints and wrists in patients with RA and reveals that significantly higher thermographic temperatures can be observed in the presence of ultrasound-detected PD as well as GS joint inflammation, although the study did not specifically look at the use of thermography for subclinical joint inflammation assessment. In another study by Gatt et al. [26] involving 31 RA patients with no clinical signs and symptoms of inflammation (of which a subset of 21 patients had no active signs of synovitis in their hands and wrist upon ultrasound assessment) and 51 healthy control, thermal imaging of the hands and wrists demonstrated higher thermographic temperatures in RA patients when compared to healthy controls. Taken together, the study by Gatt et al. [26] and our current study suggest that there may be abnormal heat signature(s) present in subclinical joint disease that could potentially be picked up by thermography. Given the scarcity of data, more RA studies looking at the potential use of thermography in subclinical joint disease detection will be necessary, and ideally, thermography should be performed alongside other more established imaging modalities (such as ultrasonography and MRI) for comparative analysis.

Our study is not without limitations. Apart from the relatively small sample size, we have compared thermal imaging with ultrasound imaging in detecting subclinical joint disease at the MCPJs by using a cross-sectional study design (i.e. at a single time-point). Therefore, we do not know how useful thermal imaging may be in the detection of subclinical joint inflammation at the MCPJs over time. Additionally, our imaging study did not include any joint damage outcome(s). Hence, we do not know if thermographic findings have any impact on joint damage at the MCPJs. Therefore, future larger scale RA studies investigating the use of thermal imaging in detecting subclinical joint disease should incorporate imaging assessments at multiple time-points (i.e. using a prospective longitudinal study design), and the imaging assessments should ideally capture both joint inflammation and damage outcomes.

## Conclusion

In summary, we have shown, for the first time, that thermographic temperatures have an association with ultrasound-detected joint inflammation at clinically quiescent MCPJs. Specifically, all our studied thermographic parameters (Total Tmin, Total Tavg and Total Tmax) showed significant correlation with ultrasound-detected joint inflammation outcomes (TGS, TPD and number of joint(s) with ultrasound synovitis defined as PD ≥ 1 or GS ≥ 2). Our study has provided new insights into thermographic detection of subclinical joint inflammation at the MCPJ in patients with RA. This, we believe, is likely to pave the way for future studies investigating the potential clinical utility of thermography in the assessment of subclinical joint disease in patients with RA.

## Data Availability

The datasets used and/or analysed during the current study are available from the corresponding author on reasonable request.

## Abbreviations

ACR: American College of Rheumatology
DAS28: 28-joint disease activity score
DMARDs: Disease-modifying anti-rheumatic drugs
EULAR: European Alliance of Associations for Rheumatology
GS: Grey-scale
MCPJs: Metacarpophalangeal joints
MRI: Magnetic resonance imaging
PD: Power Doppler
PIPJ: Proximal interphalangeal joint
RA: Rheumatoid arthritis
SD: Standard deviation
Sqrt: Square root
Tavg: Average temperature
TGS: Total grey-scale
Tmax: Maximum temperature
Tmin: Minimum temperature
TPD: Total power Doppler
